# Transcriptional Response of Silkworm (*Bombyx mori*) Eggs to O_2_ or HCl Treatment

**DOI:** 10.3390/ijms17121838

**Published:** 2016-12-07

**Authors:** Jing Gong, Sha Tian, Xia Zhou, Huan Yang, Yong Zhu, Yong Hou

**Affiliations:** State Key Laboratory of Silkworm Genome Biology, College of Biotechnology, Southwest University, Chongqing 400715, China; 03gongjing@163.com (J.G.); tiansha1015@163.com (S.T.); zhouxiagz@163.com (X.Z.); m18183079489@163.com (H.Y.)

**Keywords:** silkworm, diapause, trehalase, RNA biogenesis, heat shock protein

## Abstract

Diapause is a common biological phenomenon that occurs in many organisms, including fish, insects, and nematodes. In the silkworm (*Bombyx mori*), diapause generally occurs in the egg stage. Treatment with O_2_, HCl, or other compounds can prevent egg diapause. Here, we characterized the transcriptomic responses of newly laid eggs treated with O_2_ or HCl. Digital gene expression analysis showed that 610 genes in O_2_-treated eggs and 656 in HCl-treated eggs were differentially expressed. Of these, 343 genes were differentially expressed in both treatments. In addition to trehalases, sorbic acid dehydrogenases, and some enzymes involved in the carbohydrate metabolism, we also identified heat shock proteins, cytochrome P450, and GADD45, which are related to stress tolerance. Gene ontology enrichment analysis showed differentially expressed genes in O_2_-treated eggs were involved in oxidoreductase activity as well as in binding, catalytic, and metabolic processes. The Kyoto Encyclopedia of Genes and Genomes analysis showed that the pathways for ribosome biogenesis, spliceosome, and circadian rhythm were significantly enriched in HCl-treated eggs. The reliability of the data was confirmed by qRT-PCR analysis. Our results improved the understanding of the mechanism of diapause blocking in silkworm eggs treated with O_2_ or HCl and identified novel molecular targets for future studies.

## 1. Introduction

Diapause, defined as a period of arrested development and reduced energy consumption, is a strategy that helps animals survive extreme environmental conditions, such as cold, heat, and drought. Diapause occurs in both vertebrates and invertebrates [1,2]. In insects, diapause occurs at any stage of the development, including egg, larva, pupa, and adult, and the underlying biological mechanism has been studied extensively [3].

The silkworm, *Bombyx mori*, is an economically important insect with a breeding history of more than 5000 years in China. Diapause in silkworm usually occurs during the late gastrula stage of embryogenesis and is called embryonic diapause [4,5]. Most silkworm strains in northern China produce eggs that undergo diapause and hatching occurs once or twice per year, whereas in southern China, most silkworm strains do not produce diapause eggs under natural conditions and reproduce several times per year [6]. In addition to geography, the environmental conditions of egg development affect whether silkworm eggs enter diapause. For instance, *Dazao*, a bivoltine silkworm strain, lays eggs that experience diapause if they are incubated under long-day conditions (18 h light:6 h dark) at 25 °C during the egg-development stage, whereas when the eggs are incubated in continuous darkness at 15 °C, they do not experience diapause [7].

Although the mechanism of diapause has not been fully elucidated, previous molecular studies showed that the phenomenon is caused by a neuropeptide diapause hormone (DH) released from the neurosecretory cells of the subesophageal ganglion [8]. In the diapause stage, glucose is transformed to trehalose and glycerol, which help the eggs to overcome the adverse environmental conditions, whereas in the diapause termination stage, trehalose and glycerol are degraded [9].

Some conditions can prevent egg diapause. HCl treatment has been widely used in sericulture for preventing diapause-destined eggs from entering diapause; however, the underlying mechanism of this effect remains unclear. Previous studies have suggested that the termination of egg diapause is mainly caused by the presence of H^+^ or O^−^, as H_2_SO_4_ and other acids, O_2_, and hydrogen peroxide can interfere with diapause [10,11]. In addition, dimethyl sulfoxide and other substances with high alkyl chain structures can prevent diapause [12].

In order to investigate the mechanism of embryonic diapause in silkworm, large-scale screenings were performed, including genome-wide microarray and shotgun liquid chromatography–tandem mass spectrometry, combined with bioinformatics, to identify differentially expressed genes (DEGs) in diapause and non-diapause eggs [13,14,15]. Our goal was to study the underlying mechanism of diapause and diapause blocking by treating diapause-destined eggs with O_2_ or HCl. The DEGs of treated and non-treated eggs were analyzed by RNA-sequencing followed by bioinformatics analysis, and the molecular impact of the two treatments is discussed.

## 2. Results

### 2.1. RNA-Sequencing and General Transcription Patterns

**Figure 1:**
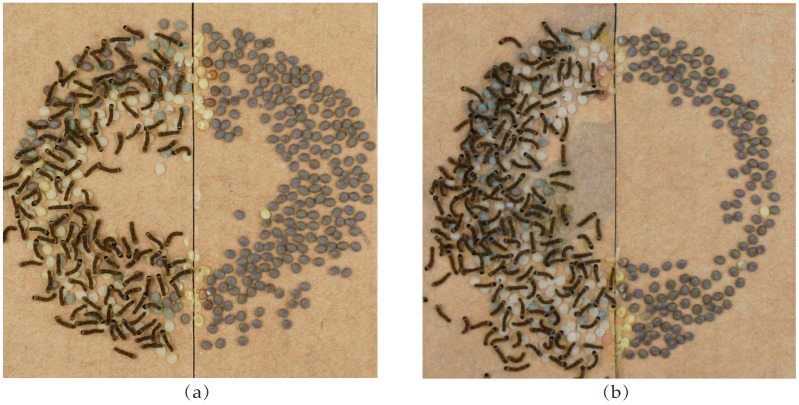
Egg incubation after different treatments. Half the eggs (**left** side) from each moth were treated with O_2_ or HCl, and the other half (**right** side) remained untreated (control). (**a**) O_2_-treated eggs and non-treated eggs and (**b**) HCl-treated eggs and non-treated eggs.

Non-treated eggs entered diapause, whereas the O_2_-treated and the HCl-treated eggs avoided diapause and developed into larva (Figure 1). In order to explore the mechanism of diapause blocking by O_2_ or HCl, we performed RNA-sequencing and obtained 12,920,255 and 11,131,963 clean reads from the O_2_-treated eggs; 13,598,605 and 13,514,169 clean reads from the HCl-treated eggs; and 12,313,326 and 12,506,248 clean reads from the non-treated eggs (Table 1). The read counts were converted to Reads Per Kilobase of exon model per Million mapped reads (RPKM) (Table S1) and showed that the distributions of gene expression levels were similar between the treated and non-treated eggs (Figure S1). The proportion of total reads that mapped to the reference genome ranged from 95.67% to 96.49%, and correlation values were significantly higher between the duplicated samples than among the treatments (Figure S2).

### 2.2. Differentially Expressed Genes (DEGs) in O_2_-Treated and HCl-Treated Eggs

**Figure 2:**
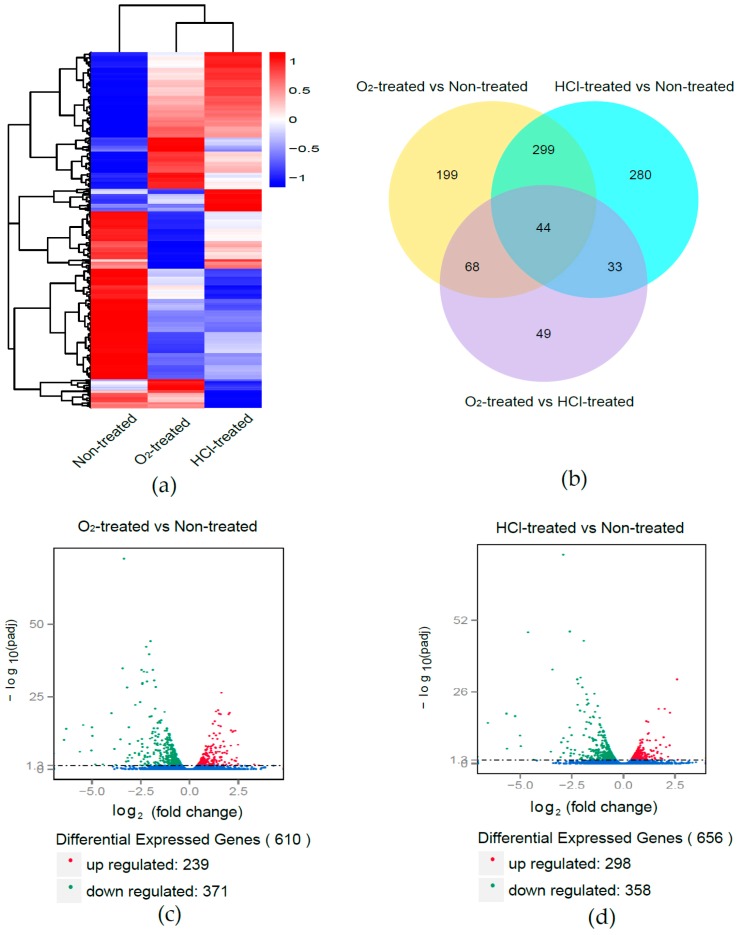
Hierarchical cluster analysis, Venn diagram, and volcano plot of differentially expressed genes (DEGs) in O_2_-treated eggs and HCl-treated eggs. (**a**) Hierarchical clustering of DEGs. Blue indicates low expression, white indicates moderate expression, and red indicates high expression. The log_10_ Reads Per Kilobase of exon model per Million mapped reads (RPKM) values were used to estimate the expression level; (**b**) Venn diagram of gene number and distribution in O_2_-treated eggs, HCl-treated eggs, and non-treated eggs; (**c**) DEGs between O_2_-treated eggs and non-treated eggs; and (**d**) DEGs between HCl-treated eggs and non-treated eggs. Red dots represent upregulated genes and green dots downregulated genes.

To observe the gene expression patterns among O_2_-treated eggs, HCl-treated eggs, and non-treated eggs, hierarchical clustering was performed based on the log_10_ RPKMs. The gene expression patterns were similar between the HCl-treated eggs and O_2_-treated eggs, but differed in the treatment groups from those of the non-treated eggs (Figure 2). In the O_2_-treated eggs, we identified 610 DEGs (239 with upregulated and 371 with downregulated expression); in the HCl-treated eggs, we identified 656 DEGs (298 with upregulated and 358 with downregulated expression). Only 194 genes showed differential expression between the HCl-treated eggs and O_2_-treated eggs (Figure 2, Tables S2 and S3).

### 2.3. Common DEGs between O_2_-Treated and HCl-Treated Eggs

**Figure 3:**
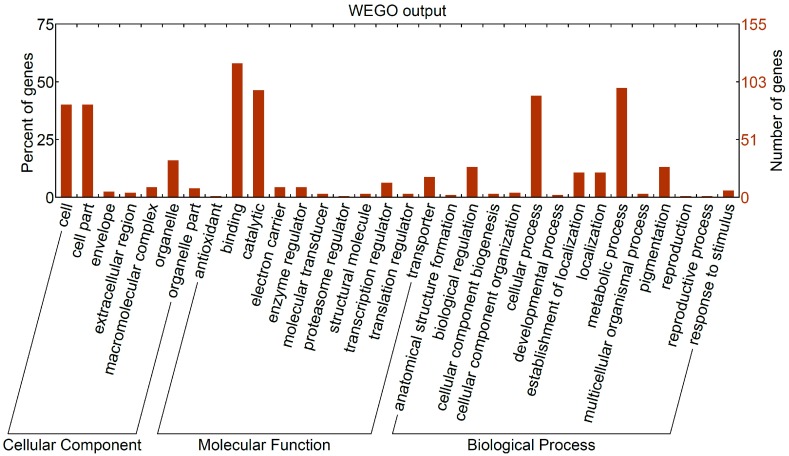
Gene Ontology (GO) categories of common differentially expressed genes (DEGs) between O_2_-treated eggs and HCl-treated eggs. The Web Gene Ontology Annotation Plot (WEGO) was used to plot GO annotation.

A total of 343 DEGs (109 upregulated and 232 downregulated) were common to both treatments, accounting for 56.2% of DEGs in O_2_-treated eggs and 52.3% of DEGs in HCl-treated eggs (Table S4). Gene Ontology (GO) analysis showed that the common DEGs were mainly distributed in the binding and catalytic terms of the molecular function category and the cellular and metabolic processes of the biological process category (Figure 3). Of the 180 genes annotated successfully by GO, 25 genes were involved in nucleic acid binding (GO:0003676), 22 genes in zinc ion binding (GO:0008270), 16 genes in ATP binding (GO:0005524), and 11 genes in oxidoreductase activity (GO:0016491) (Table S4).

### 2.4. GO and KEGG Enrichment Analysis of DEGs

**Figure 4:**
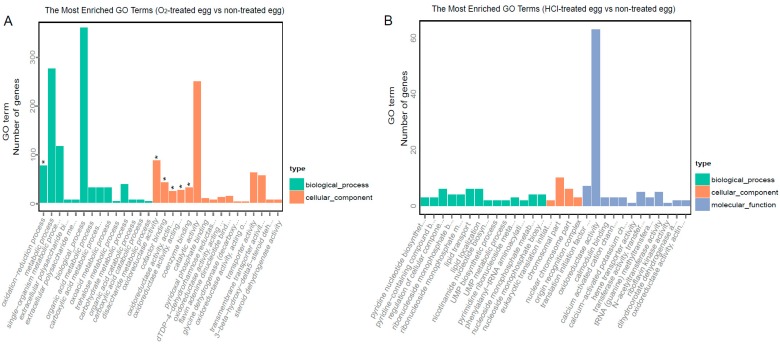
Gene Ontology (GO) enrichment of differentially expressed genes (DEGs) in (**A**) O_2_-treated eggs and (**B**) HCl-treated eggs. The statistical significance of functional GO Slim enrichment was analyzed using GOseq. The terms of oxidation-reduction process, oxidoreductase activity, and cofactor binding were significantly enriched in O_2_-treated eggs. The symbol of * indicate the significant enrichment at *p* < 0.05 during Gene Ontology analysis.

A total of 734 genes (79.52% of all DEGs) were annotated successfully by GO analysis. In the O_2_-treated eggs, significantly enriched genes were associated with cofactor binding and oxidoreductase activity in the molecular function category. In the HCl-treated eggs, no significantly enriched terms were identified, although 63 genes were involved in oxidoreductase activity in the molecular function category (Figure 4, Table S5).

### 2.5. Validation of RNA-seq Data by qRT-PCR

**Figure 5:**
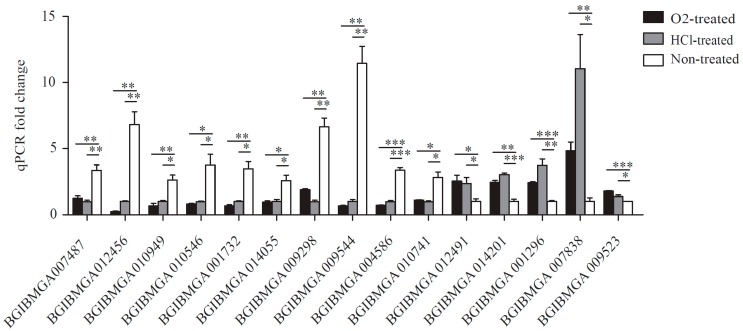
Quantitative real-time polymerase chain reaction of differentially expressed genes (DEGs) in O_2_-treated eggs and HCl-treated eggs. The relative gene expression was normalized against the housekeeping gene tif-4A (BGIBMGA003186). *, significant at *p* < 0.05; **, significant at *p* < 0.01; and ***, significant at *p* < 0.001.

The 15 DEGs that were selected randomly for qRT-PCR validation. In general, the patterns of upregulation and downregulation were consistent with those obtained from digital gene expression (DGE) analysis, confirming that both methods were reliable (Figure 5, Table S6).

## 3. Discussion

### 3.1. Trehalose Synthesis and Transportation

Trehalase and trehalose metabolism are involved in the diapause mechanism of insects. During the developmental embryonic stage of diapause eggs, diapause hormones increase trehalase activity in the developing ovaries, thus enhancing the incorporation and concentration of glucose in the oocytes [16]. Two forms of trehalase were identified in silkworm (Table S7): treh-1, a soluble protein in the cavity of goblet cells in the midgut, and treh-2, a transmembrane protein located in the membrane of follicle cells, responsible for the hydrolysis of trehalose to glucose [17,18,19]. Our results showed that treh-1 (BGIBMGA005665) was upregulated in the O_2_-treated egg compared with the non-treated eggs, whereas treh-2 (BGIBMGA004586) was downregulated in the O_2_-treated eggs and HCl-treated eggs (Table S7). After oviposition, the expression of the two types of trehalase was moderate in the mature non-treated eggs, but it exhibited significant changes in the O_2_-treated and HCl-treated eggs, suggesting that they were involved in diapause after oviposition. Furthermore, conflicting responses to O_2_ treatment indicated that treh-1 and treh-2 might have different functions in the diapause process in the silkworm egg.

Two alpha-trehalose-phosphate synthases (BGIBMGA005181 and BGIBMGA005182), known to be involved in the synthesis of trehalose [20], were identified in the silkworm egg (Table S7). Compared with the O_2_-treated and HCl-treated eggs, the diapause-destined eggs contained upregulated trehalose-phosphate synthases. Trehalose reportedly protects the integrity of cells against a variety of environmental stresses, such as dehydration, heat, and cold [20]. Therefore, the upregulation of alpha-trehalose-phosphate synthase might contribute to the synthesis of trehalose in the diapause-destined eggs.

Facilitated trehalose transporters bind to trehalose and transport it into the cell [21,22]. We identified eight facilitated trehalose transporters among the DEGs (Table S7) and classified them into two groups. The first group included BGIBMGA002635, BGIBMGA014055, BGIBMGA010741, and BGIBMGA005605, which were downregulated in the treated eggs compared with the non-treated eggs. The second group included BGIBMGA009376, BGIBMGA004566, BGIBMGA010730, and BGIBMGA003739, which were upregulated in the treated eggs compared with the non-treated eggs. However, the role of facilitated trehalose transporters in the diapause of mature silkworm eggs remains unclear.

### 3.2. Proteins Involved in Polyols

During silkworm egg diapause, glycogen is transformed into sorbitol or glycerol, which protect the embryo from unfavorable environmental conditions [23]. When diapause is terminated by cold treatment, the amount of glycogen increases progressively. NAD-dependent sorbitol dehydrogenase is a key enzyme involved in sorbitol degradation at the end of silkworm egg diapause [24]. Compared with the diapause egg, non-diapause eggs displayed higher sorbitol dehydrogenase activity during early embryonic development [24]. Temperature stress and HCl treatment can increase the expression of sorbitol dehydrogenase [13,25]. In the present study, two sorbitol dehydrogenases (BGIBMGA012399 and BGIBMGA012400) were significantly upregulated at 12 h after the O_2_ treatment (Table S8), but not after the HCl treatment, compared with the control. However, sorbitol dehydrogenase might be expressed at a later time after HCl treatment. Thus, further study is needed.

Other polyols, such as mannitol and inositol, also enhance tolerance to environmental stress. Higher levels of inositol and mannitol reportedly accumulated in the diapause spider mite and enhanced cold tolerance [26]. In the present study, several enzymes related to inositol were identified in the silkworm egg, such as inositol-triphosphate 3-kinase (BGIBMGA009298), multiple inositol polyphosphate phosphatase 1 (BGIBMGA006993), and GPI inositol-deacylase (BGIBMGA007063). All these enzymes showed differential expression in the O_2_-treated and HCl-treated eggs compared with the non-treated eggs.

### 3.3. Heat Shock Proteins (HSPs)

Heat Shock Proteins (HSPs) are known as stress proteins and molecular chaperones. Under adverse environmental conditions, HSPs are rapidly, continuously synthesized in insects and are involved in egg and pupa diapause in some species [27,28]. The four major families of HSPs that have been identified in insects are Hsp90, Hsp70, Hsp60, and the small HSPs (sHsps) [29]. sHSPs play a crucial role in stress diapause through the binding protein substrate from degradation. The expression of Hsp-12.2–like (BGIBMGA013545) in non-treated eggs was higher than that in O_2_-treated or HCl-treated eggs and its expression also was reported to be increased by heat shock stress [30]. In *Artemia franciscana*, p26 is considered necessary for survival under stress conditions and the maintenance of diapause, as encysted p26-deficient embryos terminate diapause more readily than do p26-containing embryos [31]. In addition, HSP70 (BGIBMGA004614 and BGIBMGA014618), HSP60 (BGIBMGA007349), and HSP83 (BGIBMGA004612) were upregulated in O_2_-treated and HCl-treated eggs (Table S9). When diapause terminates, proteins may be released from sHsps, then refolded with Hsp70 and probably with HSP60 and HSP90, meeting the needs of egg development.

### 3.4. Cytochrome P450 (CYP)

In insects, CYPs catalyze the metabolism of physiologically important endogenous compounds, including juvenile hormones, ecdysteroids, and pheromones, as well as being responsible for the detoxification of plant allelochemicals and insecticides [32,33]. A gene of *cyp* was also identified as a differentially expressed gene between the diapausing and post-diapausing larvae of the wild silkmoth [34]. Insect CYP sequences can be distinguished in four major clades, including the CYP2 clade, the CYP3 clade, the CYP4 clade and the mitochondrial P450 clade [35]. In the present study, nine CYPs were differentially expressed in the O_2_-treated or HCl-treated eggs compared with the non-treated eggs (Table S10). CYP4G25 (BGIBMGA001162) of the CYP4 clade was significantly downregulated in the O_2_-treated and HCl-treated eggs. Three CYPs (BGIBMGA003944, BGIBMGA010854, and BGIBMGA003926) of the CYP3 clade and one (BGIBMGA009523) of the mitochondrial P450 clade were upregulated in the O_2_-treated eggs. In addition, two CYPs (BGIBMGA013237 and BGIBMGA006916) were upregulated, whereas another two (BGIBMGA000640 and BGIBMGA001004) were downregulated, in the HCl-treated eggs. The CYP3 clade is known to be involved in the xenobiotic metabolism and insecticide resistance of *Drosophila melanogaster* and other insects [35]. Our results showed that four CYPs of the CYP3 clade were upregulated in treated eggs. As an embryo is not likely to require detoxification enzymes when it is isolated from the environment by extra-embryonic membranes, we infer that CYPs are associated with other activities, such as the electron transport respiratory chain [36].

### 3.5. Growth-Arrest and DNA Damage Inducible 45 (GADD45)

Growth-Arrest and DNA Damage Inducible 45 (GADD45) plays an important role in DNA repair, cell cycle arrest/cellular senescence/apoptosis, and stem cell maintenance [37]. In *D. melanogaster*, D-GADD45 is related to lifespan, and its expression is activated by stress factors such as oxidative stress, heat shock, and starvation [38]. It was over-expressed in *Aedes albopictus* during diapause induction [39]. Our results showed that GADD45 (BGIBMGA013938) was highly expressed in diapause-destined eggs (Tables S2 and S3), suggesting that it contributes to the resistance to external adverse conditions offered by diapause.

### 3.6. Ribosome Biogenesis and Spliceosome

**Figure 6:**
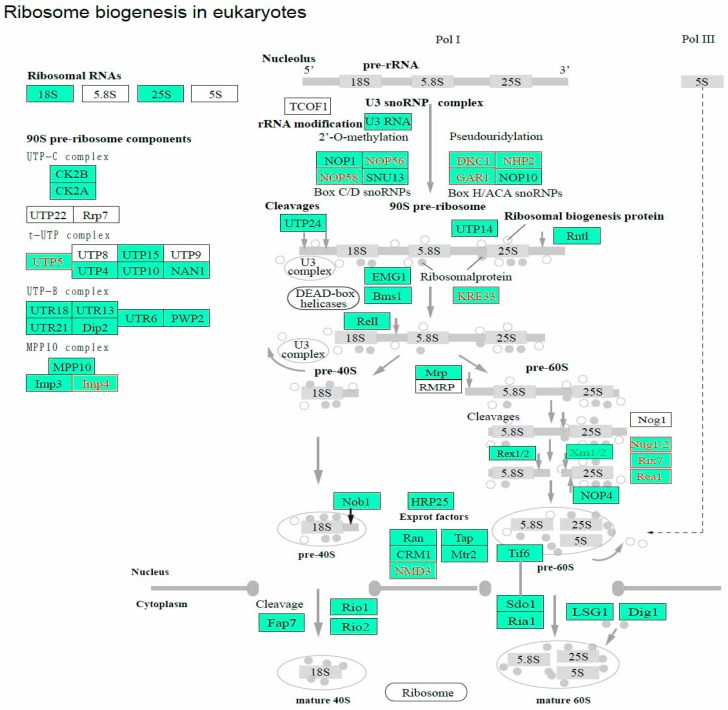
Ribosome biogenesis in eukaryote pathways constructed by Kyoto Encyclopedia of Genes and Genomes (KEGG) analysis. Red represents upregulation and green represents downregulation. A total of 12 genes were upregulated in HCl-treated eggs.

Ribosome biogenesis is linked to fundamental cellular processes, including growth and cell division [40]. In the present study, we identified 13 DEGs involved in ribosome biogenesis, of which, 12 were upregulated in the HCl-treated eggs (Figure 6, Table S11). Compared with diapause eggs, non-diapause eggs have larger nucleoli and a faster rate of ribosome synthesis during the early developmental stages after oviposition [41]. In the mosquito, *Culex pipiens*, ribosomal proteins are considered to play a role in diapause [42], and the shut-down of the ribosomal protein S3a by RNA interference produces a non-diapause mosquito that mimics the diapause state [43]. Therefore, the activity of ribosomes is characteristic in silkworm egg development, and the upregulation of ribosomal proteins plays an important role in blocking diapause.

A spliceosome is a complicated and dynamic molecular machine that composes small nuclear RNAs and ribonucleoproteins, which are responsible for the RNA splicing [44]. We identified 15 DEGs involved in the spliceosome pathway, of which, 13 were upregulated in the HCl-treated eggs (Table S12). Previous studies showed that RNA activity is important in diapause and egg development [14,45,46]; however, the underlying mechanism remains unclear and needs further investigation.

## 4. Materials and Methods

### 4.1. Sample preparation

Bivoltine silkworms (*Dazao*) were provided by the State Key Laboratory of Silkworm Genome Biology. Diapause-destined eggs were obtained from female moths that had been incubated under long-day conditions (18 L:6 D) at 25 °C and 75% relative humidity during embryonic development. The silkworm eggs from different matings were collected and treated separately. In order to prevent egg entering into diapause, at 20 h after oviposition, half the eggs from a single moth were treated with high-purity O_2_ of 70% concentrations in the mutigas incubator (Heal Force, Hongkong, China) for 40 h or with HCl solution (density = 1.075 g/mL) for 70 min. The eggs were then washed with H_2_O to remove HCl. The other half of the eggs remained untreated (control). For DGE analysis, samples from O_2_-treated eggs, HCl-treated eggs, and non-treated eggs were collected at 32 h after oviposition, immediately frozen in liquid nitrogen, and stored at −80 °C until use. Two samples were collected from each treatment group.

### 4.2. RNA Sequencing

Total RNA was extracted using TRIzol reagent (Invitrogen, Waltham, MA, USA). RNA purity was monitored by a spectrophotometer, and RNA degradation was analyzed by 1.5% agarose gel electrophoresis. The cDNA library was constructed using the TruSeq RNA Sample Preparation Kit (Illumina, San Diego, CA, USA), following the manufacturer’s protocol. cDNA samples were clustered and sequenced by the Illumina HiSeq 2000 platform (Illumina). The raw data were submitted to the National Center for Biotechnology Information Short Read Archive (NCBI-SRA; http://www.ncbi.nlm.nih.gov/sra/) under the accession number PRJNA327613.

### 4.3. Data Analysis

The raw data were processed by Perl scripts to remove adapter sequences and low-quality reads. The reference genome was downloaded from the Silkworm Genome Database (ftp://ftp.ensemblgenomes.org/pub/release-23/metazoa/fasta/bombyx_mori/dna/) and built using Bowtie 2.0.6 [47]. The clean reads were aligned to the reference genome using TopHat [48]. The sequencing data were analyzed using the CLC Genomics Workbench (Qiagen, Valencia, CA, USA), following the manufacturer’s protocol [49]. HTSeq (http://www.huber.embl.de/users/anders/HTSeq) was used to calculate the reads mapped to each transcript, and the reads per kilobase of exon model per million mapped reads (RPKM) method was used to quantify gene expression (if RPKM ≥ 1, a gene is expressed in a sample). The read counts were calculated to analyze the significant differential expression using edgeR [50], whereas DEG analysis was performed using DEGSeq R (TNLIST, Beijing, China). Differential expression was considered significant at *p* < 0.05.

### 4.4. GO Annotation and Enrichment Analysis

Blast2GO was employed to annotate the DGE functions using InterProScan (http://www.ebi.ac.uk/interpro/) or BLASTX against the NCBInr database, followed by Gene Ontology (GO) annotations. The statistical significance of the functional GO Slim enrichment was performed using GOseq [51]. The Web Gene Ontology Annotation Plot (WEGP; http://wego.genomics.org.cn/cgi-bin/wego/index.pl) was used to plot the GO annotation results of the common DEGs between O_2_-treated and HCl-treated eggs and the Molecular Annotation System (MAS) 3.0 (http://bioinfo.cau.edu.cn/agriGO) to identify their category. In order to explore the biological interaction among DEGs, the Kyoto Encyclopedia of Genes and Genomes (KEGG) pathways were investigated and enriched by KOBAS 2.0 (http://kobas.cbi.pku.edu.cn/home.do) with the hypergeometric test and the Benjamini-Hochberg false discovery rate (FDR) correction [52].

### 4.5. Validation of DEGs by Quantitative Real-Time Polymerase Chain Reaction (qRT-PCR)

A total of 15 genes was randomly selected for qRT-PCR validation. Total RNA was extracted using TRIzol (Invitrogen) and reverse transcribed to cDNA by M-MLV reverse transcriptase (Promega, Madison, WI, USA). The primers were designed using Primer Premier 5.0 (Biosoft, Palo Alto, CA, USA) and synthesized by BGI (Shenzhen, China) (Table S13). The housekeeping gene tif-4A (BGIBMGA003186) was selected as an internal control. qRT-PCR was performed using the Real-Time PCR Detection System (Bio-Rad Laboratories, Berkeley, CA, USA) with 10 μL SYBR Premix Ex Taq (TaKaRa, Osaka, Japan), 2 μL cDNA, and 0.2 μM of each primer. The thermo-cycling parameters were as follows: initial denaturation for 30 s at 95 °C and 40 cycles of 3 s at 95 °C and 30 s at 60 °C. Each sample was analyzed in triplicate.

## 5. Conclusions

We used O_2_ and HCl treatment to prevent facultative diapause eggs from entering diapause. DGE analysis at 12 h after treatment showed that proteins involved in egg diapause or egg development, such as trehalases, trehalose transporters, sorbitol dehydrogenases, and HSPs, displayed a differential expression pattern in O_2_-treated or HCl-treated eggs and non-treated eggs. These data provide new insights into the mechanism of silkworm egg diapause and the effects of O_2_ or HCl on the prevention of egg diapause. In our future studies, we aim to investigate the developmental changes between treated and non-treated eggs using metabolomics in order to analyze the production and function of metabolites during egg diapause and development.

## Figures and Tables

**Table 1:** Statistical analysis of DEGseq data obtained from O_2_-treated eggs and HCl-treated eggs.

Sample Name	Raw Reads	Clean Reads	Clean Bases	Total Mapped	Error Rate (%)	Q20 (%)	Q30 (%)	GC Content (%)
O_2_-treated_1	12,984,364	12,920,255	0.65 G	12,360,505 (95.67%)	0.01	97.9	94.07	43.13
O_2_-treated_2	11,192,761	11,131,963	0.56 G	10,740,908 (96.49%)	0.01	98.84	96.07	43.38
HCl-treated_1	13,670,896	13,598,605	0.68 G	13,068,581 (96.1%)	0.01	97.92	94.13	43.44
HCl-treated_2	13,595,227	13,514,169	0.68 G	12,993,548 (96.15%)	0.01	98.64	95.87	45.03
Non-treated_1	12,361,440	12,313,326	0.62 G	11,827,559 (96.05%)	0.01	97.91	94.12	43.15
Non-treated_2	12,559,734	12,506,248	0.63 G	11,993,516 (95.9%)	0.01	98.16	94.66	43.5

## Supplementary Materials

Supplementary materials can be found at www.mdpi.com/1422-0067/17/12/1838/s1.

## Abbreviations

HSP	Heat shock protein
DGE	Digital gene expression
DEGs	Differentially expressed genes
