# Supplementary material for: Transcriptional Response of Silkworm (Bombyx mori) Eggs to O2 or HCl Treatment
Source: Int J Mol Sci. 2016 Dec 7;17(12):1838. doi: 10.3390/ijms17121838 (PMC5187757; doi:10.3390/ijms17121838)
Supplement: Supplementary file 1 [file ijms-17-01838-s001.zip › ijms-152376-Supplementary Materials/ijms-152376-si-figure.pdf]

Supplementary Materials: Transcriptional Response of Silkworm (*Bombyx mori*) Eggs to O_2 or HCl Treatment

Jing Gong, Sha Tian, Xia Zhou, Huan Yang, Yong Zhu and Yong Hou

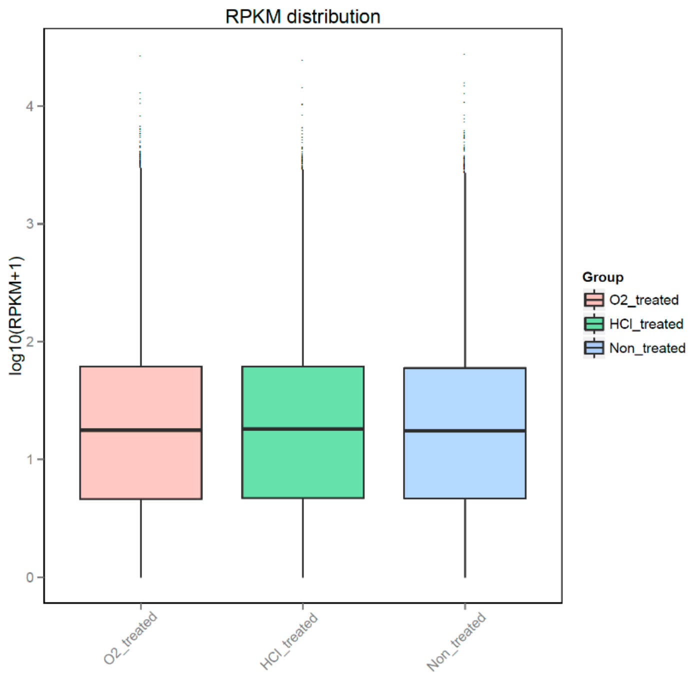

Figure S1. Boxplot of the log10 of Reads Per Kilobase of exon model per Million mapped reads (RPKM) values of O2-treated eggs, HCl-treated eggs, and non-treated eggs. The solid horizontal line indicates the median and the box the lower and upper quartiles.

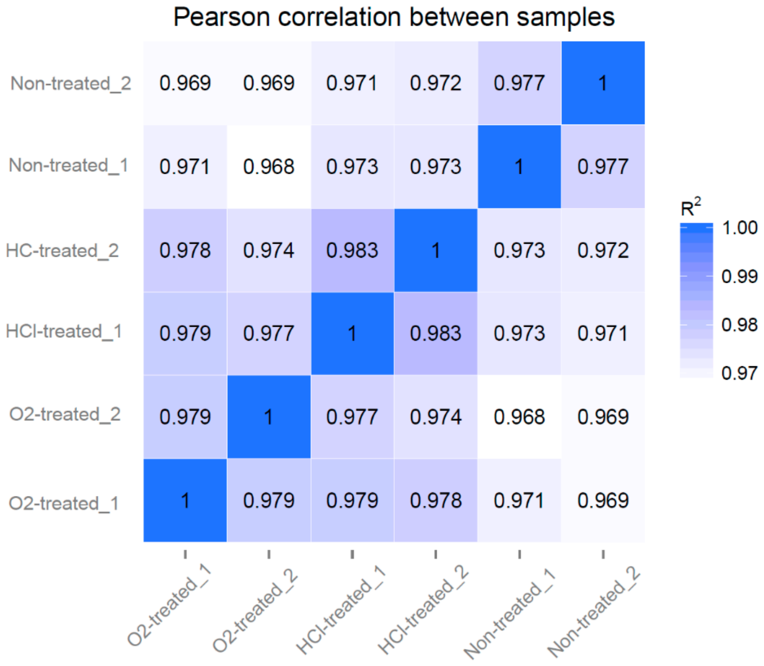

Figure S2. Pearson correlation between O2-treated eggs, HCl-treated eggs, and non-treated eggs.cripts/upload/799c28dc699c1a65d0b2753c58894687/5.
